# Lycopene Improves Bone Quality and Regulates AGE/RAGE/NF-кB Signaling Pathway in High-Fat Diet-Induced Obese Mice

**DOI:** 10.1155/2022/3697067

**Published:** 2022-02-17

**Authors:** Bingke Xia, Ruyuan Zhu, Hao Zhang, Beibei Chen, Yage Liu, Xuan Dai, Zimengwei Ye, Dandan Zhao, Fangfang Mo, Sihua Gao, Xiang-Dong Wang, Dieter Bromme, Lili Wang, Xinxiang Wang, Dongwei Zhang

**Affiliations:** ^1^Diabetes Research Center, Traditional Chinese Medicine School, Beijing University of Chinese Medicine, Beijing 100029, China; ^2^Jean Mayer USDA Human Nutrition Research Center on Aging at Tufts University, Boston, MA 02111, USA; ^3^Department of Oral Biological & Medical Sciences, Faculty of Dentistry, The University of British Columbia, Vancouver, BC, Canada V6T 1Z3; ^4^Department of TCM Pharmacology, Chinese Material Medica School, Beijing University of Chinese Medicine, Beijing 102488, China; ^5^The Scientific Research Center, Dongfang Hospital, Beijing University of Chinese Medicine, Beijing 100078, China

## Abstract

**Objective:**

This study was aimed at examining the effects of lycopene on bone metabolism in high-fat diet (HFD)- induced obese mice and to identify the potential underlying mechanisms.

**Methods:**

Mice were fed a HFD for 12 weeks and then continue with or without lycopene intervention (15 mg/kg) for additional 10 weeks. The effects of lycopene on blood glucose and lipid metabolism, as well as serum levels of total antioxidant capacity (T-AOC), superoxide dismutase (SOD), and malondialdehyde (MDA) were determined by biochemical assays. Bone histomorphological features and osteoclast activity were assessed by hematoxylin/eosin and tartrate-resistant acid phosphatase staining. Bone microstructure at the proximal tibial metaphysis and diaphysis was determined by microcomputed tomography. Tibial biomechanical strength and material profiles were measured by a three-point bending assay and Fourier transform infrared spectroscopy. Protein expressions involved in the AGE/RAGE/NF-кB signaling pathway were determined by western blot and/or immunohistochemical staining.

**Results:**

Lycopene consumption reduced body weight gain and improved blood glucose and lipid metabolism in HFD-induced obese mice. In addition, lycopene treatment preserved bone biomechanical strength, material profiles, and microarchitecture in obese mice. Moreover, these alterations were associated with an increase in serum levels of T-AOC and SOD, and a decline in serum levels of MDA, as well as a reduction of AGEs, RAGE, cathepsin K, and p-NF-кBp65 and NF-кBp65 expressions in the femurs and tibias of obese mice.

**Conclusion:**

Lycopene may improve bone quality through its antioxidant properties, which may be linked with the regulation of the AGE/RAGE/NF-кB signaling pathway in obese mice. These results suggest that lycopene consumption may be beneficial for the management of obesity-induced osteoporosis.

## 1. Introduction

As a metabolically active tissue, bone is regulated by systemic glucose and lipid metabolism [1]. Given the worldwide prevalence of obesity and hyperglycemia, the study of bone metabolism has gained increasing attention [2–4]. Accumulating evidence suggests that sustained dyslipidemia and hyperglycemia impair bone microstructure, material compositions, and biomechanical strength, leading to an increased risk of bone fractures [5–7]. However, clinical studies revealed that bone quality is mostly neglected during obesity management [8, 9]. In addition, current pharmacological countermeasures for obesity and osteoporosis are associated with poor adherence and adverse complications [10]. Emerging evidence suggests that dietary intervention may provide a new strategy for the effective management of bone and lipid metabolism [11–13].

Advanced glycation end products (AGEs), mainly derived from sustained hyperglycemia, drive oxidative stress generation and abnormal bone remodeling [14, 15]. AGEs may promote osteoclastic bone resorption and inhibit osteoblastic bone formation through the regulation of the receptor for AGEs (RAGE) [16]. In addition, AGEs compromise bone quality and increase fracture risk through altering bone material constituents [17]. Moreover, elevation of AGEs enhances the production of reactive oxygen species (ROS), which further triggers oxidative stress via RAGE. These interactions result in the activation of nuclear factor-kappa B (NF-кB) translocation and promote subsequent cathepsin K (catK) expression, leading to increased bone resorption [18, 19]. As has been shown previously, mice deficient in RAGE exhibit a decline in osteoclasts and an increase in bone mass [20]. In addition, our previous studies suggested that the inhibition of AGEs/RAGE/NF-кB and NADPH oxidase 4/ROS/NF-кB signaling improves bone quality in diabetic and ovariectomized rats [16, 18]. Bucala et al. also showed that the administration of an advanced glycation inhibitor to diabetic subjects helps to prevent dyslipidemia [21]. Thus, the inhibitions of AGEs and ROS generation may offer a new strategy of protection against metabolic syndrome-related derangements involving bone tissue.

Lycopene, a dietary carotenoid, mainly found in tomatoes and other red colored fruits [19], is well known for its high antioxidant potential [22]. There are accumulating evidences suggesting that lycopene consumption helps to improve bone metabolism [23–26]. We and others have demonstrated that lycopene could improve blood glucose and lipid metabolism in preclinical studies and clinical trials [27, 28]. However, the possibility that lycopene administration may prevent bone loss in patients with glucose and lipid metabolism disorders remains unexplored. Therefore, our aim for the present study is to examine the effects of lycopene on bone quality in high-fat diet (HFD)- induced obese mice, as well as its associations with the AGE/RAGE/NF-кB signaling pathway.

## 2. Materials and Methods

### 2.1. Reagents and Antibodies

Lycopene was purchased from RuiFenSi Biotechnology Co., Ltd. (Chengdu, China). The glucose consumption assay kit was obtained from Applygen Technologies Inc. (Beijing, China). Antigen retrieval solution was bought from ShunBai Biotechnology Company (No. SBT10013; Shanghai, China). Total antioxidant capacity (T-AOC), superoxide dismutase (SOD), and malondialdehyde (MDA) kits were purchased from Nanjing Jiancheng Bioengineering Institute (Nanjing, China). Antibodies against AGEs (ab23722), RAGE (ab37647), and catK (ab19027) were from Abcam Biocompany (Cambridge, MA, USA). Antibodies against p-NF-кBp65 (WL02169) and NF-кBp65 (WL01980) were from Wanlei Biotechnology (Shenyang, China). Antibodies against GAPDH (60004-1-Ig) and *β*-actin (66009-1-Ig) were from Proteintech Biotechnology (Wuhan, China). HRP-conjugated secondary antibodies were procured from Proteintech Biotechnology. All other reagents excluding those previously identified were from Sinopharm Reagents Co. Ltd (Beijing, China).

### 2.2. Animals

Male Institute of Cancer Research (ICR) mice weighing about 20 ± 2 g were bought from Beijing SiBeiFu Animal Technology Co. Ltd. (Beijing, China) and housed in the specific-pathogen-free (SPF) animal facility with a constant temperature (22°C ± 2°C) and humidity (55% ± 5%) and a 12-h light/dark cycle at the Beijing University of Chinese Medicine (BUCM). All mice were supplied with regular chow and drinking water *ad libitum*. All the animal procedures were approved by the BUCM Animal Care Committee, Beijing, China.

### 2.3. Diet-Induced Obesity and Lycopene Administration

After acclimation for 1 week, the ICR mice were fed a HFD (45% fat, Rodent Diet D12032; Research Diet, Jiangshu, China) for 12 consecutive weeks. The HFD-fed mice that met the following requirements were enrolled in the subsequent experiments [29]: (1) body weight gain greater than 20% and (2) a fasting glucose level over 7.8 mM. The eligible mice were randomly divided into three groups and treated for additional 10 weeks as shown in Table 1. In addition, 9 mice fed a regular chow diet were used as normal controls.

During the experiment, the body weight of the mice was measured once a week. At the end of treatment after anesthesia, body weight and length were measured. Blood was harvested from the heart for further analysis. Meanwhile, the bilateral tibias and femurs were removed from the animal bodies. The samples were then collected according to the requirements for further experiments.

### 2.4. Glucose Tolerance Tests

An oral glucose tolerance test (OGTT), as previously described [29], was conducted on mice at the 20^th^ week after the lycopene supplementation for 8 weeks. The glucose levels were measured at 0, 30, 60, 90, and 120 min after oral administration of glucose (2 g/kg) using a glucometer (Contour Plus, Bayer).

### 2.5. Serum Biomarkers Analysis

Serum levels of triglycerides (TG), total cholesterol (TC), high-density lipoprotein (HDL), low-density lipoprotein (LDL), T-AOC, SOD, and MDA were determined using the commercial kits according to the manufacturer's protocols.

### 2.6. Hematoxylin/Eosin (H&E) and Tartrate-Resistant Acid Phosphatase (TRAP) Stainings

The paraffin-embedded sections (∼5 *μ*m thickness) from left femurs of the mice were processed as previously described [30]. The H&E staining was performed according to the routine procedure.

TRAP staining was conducted as follows: the deparaffinized and rehydrated sections were incubated with Naphthol AS-BI phosphate solution (basic solution) for 45 min at 37°C. After that, the slides were incubated with the basic solution with brown dyes (sodium nitrite and parafuchsin) for 6 min at room temperature followed by hematoxylin staining.

After staining, the histopathological alterations of trabecular bone were photographed and determined using an Olympus BX53 fluorescence microscope (Tokyo, Japan). The numbers of osteoclasts (OCs) were counted from the images.

### 2.7. Microcomputed Tomography (*μ*-CT) Analysis of Tibias

*μ*-CT scanning was performed on the left tibia as previously described [31]. The alterations of the proximal tibial metaphysis and diaphysis were examined. The following parameters in the volume of interest were analyzed by the Analyzer Software (V12.0) as shown in Table 2 [32, 33].

### 2.8. Bone Biomechanical Strength and Material Profile Assays

After *μ*-CT scanning, the right tibias were used for a three-point bending examination by an electronic universal testing machine (Brookfield CT3, America) as previously described [30].

After that, the tibia was triturated under liquid nitrogen in a ceramic mortar. The spectrum was obtained by a Fourier transform infrared spectroscopy (FTIR; Bruker Vertex 70, Germany). Scanning was conducted in transmission mode (4,000 - 400 cm^−1^ range, 4 cm^−1^ resolution, and 64 scans) [34]. The following parameters were determined as follows: (1) collagen maturity, the relative intensity ratios of the 1,660^−1^ to 1,690 cm^−1^ peaks [35]; (2) mineral maturity/crystallinity, the intensity ratio of 1030 cm^−1^ to 1020 cm^−1^ [36]; (3) the relative ratio of mineralization to the collagen matrix (mineral/matrix) [37]; and (4) the relative ratio of carbonate to phosphate, the area ratio v1, v3 band to v2 CO_3_ [38, 39].

### 2.9. Immunohistochemical (IHC) Analysis

IHC staining was conducted as previously described [31]. Briefly, the slides were incubated with the appropriate primary antibody [AGEs (1 : 50), RAGE (1 : 100), p-NF-кBp65 (1 : 100), NF-кBp65 (1 : 100), and catK (1 : 100)] overnight at 4°C followed by the corresponding secondary antibody. Subsequently, slides were examined and photographed using an Olympus BX53 microscopy. The intensity of DAB staining was analyzed using an Image Pro Plus6.0 software and expressed as IOD value.

### 2.10. Western Blot Assay

The protein samples were prepared and transferred to the membranes as previously described [16]. The primary antibodies were as follows: AGEs (1 : 500), RAGE (1 : 1,000), p-NF-кBp65 (1 : 500), NF-кBp65 (1 : 500), GAPDH (1 : 20,000), and *β*-actin (1 : 5,000). The images captured with Azure Bioimaging systems were quantified using the Image J software and normalized with the corresponding *β*-actin or GAPDH.

### 2.11. Statistical Analysis

Data statistical analyses and graphical representations were conducted using GraphPad Prism (version 6.0, GraphPad Software, Inc., USA). All the data were presented as mean ± SD. When the data met homogeneity of variance and normality, a one-way analysis of variance (ANOVA) was employed. A Dunnett's T3 test or nonparametric test was applied, respectively, when the data met a normal distribution, but the homogeneity of variances was not achieved, or did not meet a normal distribution. Pearson's correlation analysis was used to examine the correlations between redox indicators and bone quality markers. Statistical significance was determined at *p* < 0.05.

## 3. Results

### 3.1. Lycopene Improves Glucose and Lipid Metabolism in Obese Mice

As illustrated in Table 3, HFD exposure significantly increased body weight and fasting blood glucose levels in ICR mice. In detail, the body weight of the mice increased by ~17.2%, ~16.7%, and ~18.5%, respectively, in the groups of HFD-C, HFD + MET, and HFD + LYC relative to that of the NC group after 12 weeks' HFD exposure. Moreover, at the 22^nd^ week, the body weight of the mice in the HFD-C group increased by ~33% compared to that in the NC group. Interestingly, metformin and lycopene intervention significantly inhibited body weight gain by ~5.5% and~4.7% in obese mice (*p* < 0.05).

As for blood lipids, mice in the HFD-C group displayed obvious elevations in TG (~33%), TC (~77%), and LDL (~4-fold) and a significant decrease in HDL (~42%) relative to those in the NC group (*p* < 0.05). Notably, mice in the MET and LYC groups exhibited a relative lower level of TG (~23%), TC (~20%), and LDL (~65%) and a higher level of HDL (~42%) than those in the HFD-C group.

In addition, at the 20^th^ week, the blood glucose levels of the mice in the HFD-C group at 0, 30, 60, 90, and 120 min were higher than those in the NC group by OGTT assay (Table 4). Moreover, at the 22^nd^ week, the fasting blood glucose (FBG) levels of the mice in the HFD-C group were increased by ~53% relative to those in the NC group (Table 3). These alterations were reversed by LYC and MET intervention as evidenced by ~20% and~25% decrease in the FBG levels, respectively, in comparison with those in the HFD-C group (*p* < 0.05). Together, these results suggest that lycopene could stabilize blood glucose and lipid metabolism in obese mice.

### 3.2. Lycopene Preserves Bone Microarchitecture, Strength, and Material Profiles in Obese Mice

**Figure 1:**
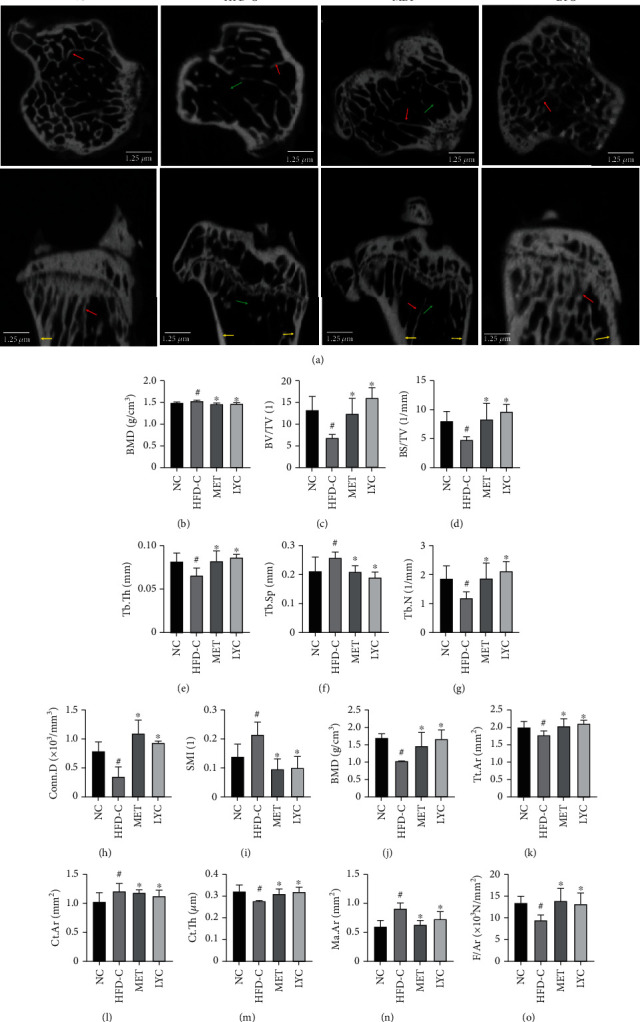
Lycopene preserves bone microarchitecture and strength in HFD-induced obese mice. The representative microimages of the transverse and longitudinal sections in the proximal tibia were obtained from micro (*μ*)-CT (the red arrow indicates trabecular bone; the green arrow indicates bone marrow cavity. The yellow arrow indicates cortical bone) (a). *μ*-CT-derived quantification data of tibial metaphysis including BMD (b), BV/TV (c), BS/TV (1/mm) (d), Tb.Th (mm) (e), Tb.Sp (mm) (f), Tb.N (1/mm) (g), Conn.D (1/mm^3^) (h), and SMI (i). The quantification data of tibial diaphysis including BMD (j), Tt.Ar (mm^2^) (k), Ct.Ar (mm^2^) (l), Ct.Th (um) (m), and Ma.Ar (mm^2^) (n). The ultimate load in the tibia, F/Ar (N/mm^2^) (o). Data are presented as mean ± SD. LYC denotes lycopene treatment; MET denotes metformin treatment. ^#^*vs* the NC group. ^∗^*vs* the HFD-C group. *p* < 0.05 was considered statistically significant.

To examine whether lycopene could protect bone microarchitecture from HFD exposure, a *μ*-CT assay was performed. As shown in Figures 1(a)–1(n), the *μ*-CT images of the proximal tibial metaphysis and its analyses demonstrated that the mice in the HFD-C group exhibited a decline in BV/TV, BS/TV, Tb.N, Tb.Th, and Conn.D, as well as an increase in BMD, Tb.Sp, and SMI when compared to those in the NC group (Figures 1(b)–1(i); *p* < 0.05). As for the tibial diaphysis, mice in the HFD-C group presented a decrease in BMD, Tt.Ar, and Ct.Th and an increase in Ct.Ar and Ma.Ar when compared to those in the NC group (Figures 1(j)–1(n); *p* <0.05). Interestingly, the administration of metformin or lycopene to obese mice reversed the aforementioned alterations of bone microstructure (*p* < 0.05) except for Ct.Ar in the tibial diaphysis.

To further evaluate the action of lycopene on bone biomechanical properties, mice tibias were subjected to the three-point bending assay. As shown in Figure 1(o), the ultimate load of the mice in the HFD-C group was notably lower than that in the NC one (*p* < 0.05). As expected, the administration of lycopene or metformin to HFD-exposed obese mice inhibited a decline in the ultimate load in the tibias (*p* < 0.05).

**Figure 2:**
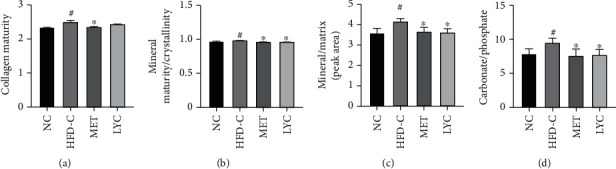
Lycopene preserves bone material profiles in HFD-induced obese mice. Collagen maturity (a), mineral maturity/crystallinity (b), collagen matrix (mineral/matrix, (c)), and carbonate to phosphate (d) were determined by Fourier transform infrared spectroscopy (FTIR). LYC denotes lycopene treatment; MET denotes metformin treatment. Data are presented as mean ± SD. ^#^*vs* the NC group. ^∗^*vs* the HFD-C group. *p* < 0.05 was considered statistically significant.

Next, we analyzed the bone material alterations, including collagen maturity, mineral maturity/crystallinity, collagen matrix (mineral/matrix), and carbonate/phosphate in the tibias by FTIR. As shown in Figures 2(a)–2(d), the collagen maturity, the relative ratios of mineral maturity to crystallinity, mineral to matrix, and carbonate to phosphate were notably raised in the HFD-C group relative to the NC group (*p* < 0.05). The treatment with lycopene prevented the alterations in the tibias of HFD-exposed mice (*p* < 0.05) except for collagen maturity. Notably, metformin treatment reversed all the alterations in the tibias of HFD-exposed ones. These results suggest that lycopene not only maintains bone strength and microstructure but also improves bone material profiles in obese mice.

### 3.3. Lycopene Improves Femoral Histopathological Alterations in Obese Mice

**Figure 3:**
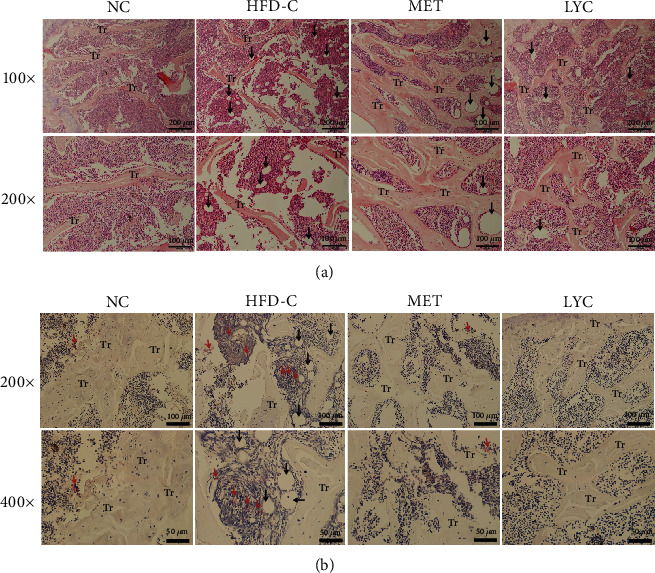
Lycopene improves bone histomorphological features in HFD-induced obese mice. Representative images of H&E (a) and TRAP (b) staining in the femoral metaphysis of different groups of mice. The black arrow in panel (a) indicates lipid droplet. The red arrow in panel (b) indicates osteoclast. Tr denotes trabecular bone. LYC denotes lycopene treatment; MET denotes metformin treatment.

To observe bone morphological alterations, H&E and TRAP stainings were performed. As illustrated in Figure 3(a), the trabecular bone in the distal femurs of the mice in the HFD-C group lost regular mesh structure and became thinner and irregular as compared to the mice in the NC group. Furthermore, the lipid droplets (indicated by the black arrow) were much more obvious in the proximal femurs of HFD-C group. As expected, lycopene or metformin treatment improved femoral histopathological alterations.

As shown in Figure 3(b), the number of OCs was identified by TRAP staining (indicated by the red arrow). The mice in the HFD-C group exhibited an increased number of OCs in the femurs (*p* < 0.05). Notably, the trend was prevented by lycopene or metformin intervention (*p* < 0.05).

### 3.4. Lycopene Alleviates Oxidative Stress in Obese Mice

**Figure 4:**
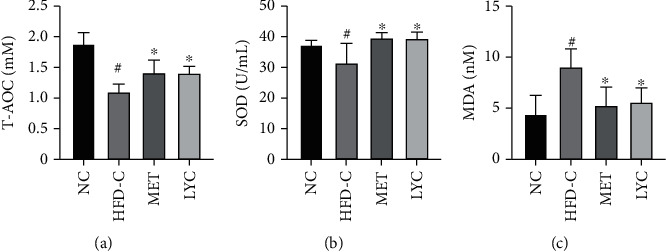
Lycopene ameliorates oxidative stress in HFD-induced obese mice. Serum levels of oxidative stress markers were determined by biochemical assays, including total antioxidant capacity (T-AOC, (a)), superoxide dismutase (SOD, (b)), and malondialdehyde (MDA, (c)). LYC denotes lycopene treatment; MET denotes metformin treatment. Data are presented as mean ± SD. ^#^*vs* the NC group. ^∗^*vs* the HFD-C group. *p* < 0.05 was considered statistically significant.

As shown in Figures 4(a)–4(c), serum levels of T-AOC and SOD were diminished, and serum levels of MDA were elevated in the mice of the HFD-C group, respectively, as compared to those in the NC group (*p* < 0.05). Interestingly, lycopene or metformin supplementation reversed these HFD-induced alterations in obese mice (*p* < 0.05). These results indicate that lycopene may alleviate oxidative stress in obese mice.

In addition, the correlation between blood markers and bone quality parameters was analyzed using Pearson correlation analysis. As shown in Table 5, the results showed that bone quality parameters were negative correlated with serum levels of TG, TC, LDL, and MDA and positively correlated with serum levels of HDL, SOD, and T-AOC. In detail, there were significant positive correlations between TG, TC, T-AOC, and BMD of proximal tibial metaphysis, between Tb.N, SMI, and BMD of tibial diaphysis and SOD, between Ma.Ar of tibial diaphysis and TG, and between SMI and T-AOC. In addition, there were significant negative correlations between BV/TV, Tb.N, BMD of tibial diaphysis, and MDA. These results suggested that blood glucose and lipid metabolism could affect bone quality.

### 3.5. Lycopene Inhibits the Expressions of AGEs, RAGE, NF-кB, and catK in the Femurs and Tibias of Obese Mice

**Figure 5:**
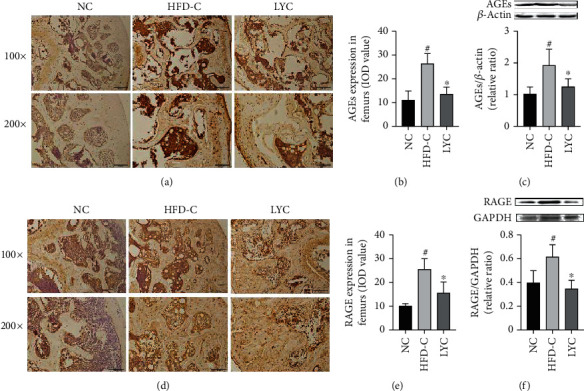
Lycopene inhibits the expressions of AGEs and RAGE in the femurs and tibias of HFD-induced obese mice. The expressions of AGEs and RAGE were analyzed by immunohistochemical staining (a, b, d, and e) and western blot (c and f). LYC denotes lycopene treatment; MET denotes metformin treatment. Data are presented as mean ± SD. ^#^*vs* the NC group. ^∗^*vs* the HFD-C group. *p* < 0.05 was considered statistically significant.

Persistent hyperglycemia results in an over-accumulation of AGEs, which enhance ROS generation and contribute to NF-кB activation, ultimately leading to bone resorption via RAGE [40, 41]. As shown in Figures 5(a)–5(f), the expressions of AGEs and RAGE in the femurs and tibias were notably increased in the mice of the HFD-C group when compared to those in the NC group (*p* < 0.05). And lycopene treatment significantly reduced the expressions of AGEs and RAGE in the hindlimbs of obese mice compared to those of the vehicle-treated controls (*p* < 0.05).

**Figure 6:**
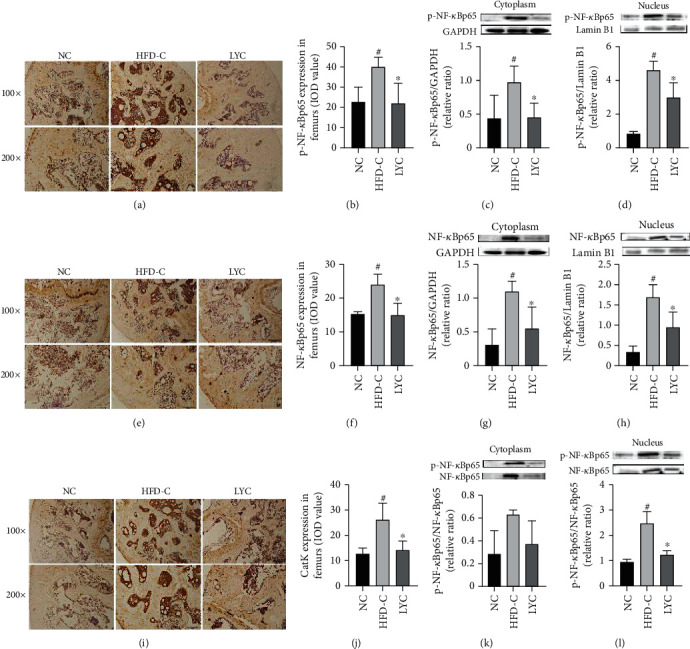
Lycopene suppresses the expressions of p-NF-кBp65, NF-кBp65, p-NF-кBp65/NF-кBp65, and catK in the femurs and tibias of HFD-induced obese mice. The expressions of p-NF-кBp65, NF-кBp65, and catK and the relative ratio of p-NF-кBp65 to NF-кBp65 were determined by immunohistochemical staining (a, b, e, f, e, and j) and western blot (c, d, g, h, k, and l). LYC denotes lycopene treatment; MET denotes metformin treatment. Data are presented as mean ± SD. ^#^*vs* the NC group. ^∗^*vs* the HFD-C group. *p* < 0.05 was considered statistical difference.

Moreover, as shown in Figures 6(a)–6(l), the expressions of NF-кBp65, p-NF-кBp65, and catK, as well as the nuclei ratios of p-NF-кBp65 to NF-кBp65 were significantly increased in the femurs and tibias of the HFD-C group of mice compared with those in the NC group (*p* < 0.05). Intriguingly, lycopene treatment markedly reduced the expressions of NF-кBp65, p-NF-кBp65, catK, and the nuclei ratio of p-NF-кBp65 to NF-кBp65 in the tibias and femurs of obese mice (*p* < 0.05). However, there were no differences in the relative ratios of p-NF-кBp65 to NF-кBp65 in the cytoplasm of the bones amongst the three groups. These results suggest that lycopene may regulate the AGE/RAGE/ROS/NF-кB signaling pathway in HFD-induced obese mice.

## 4. Discussion

In the present study, we demonstrated that lycopene reduces body weight gain and improves blood glucose and lipid metabolism in obese mice. Secondly, lycopene treatment reverses obesity-induced alterations in the bone microarchitecture, biomechanical strength, and material profiles. Thirdly, lycopene intervention increases serum levels of T-AOC and SOD and decreases serum levels of MDA in obese mice. In addition, we provided the evidence of lycopene improving bone metabolism by inhibiting obesity-induced upregulation of AGEs, RAGE, p-NF-кBp65, NF-кBp65, p-NF-кBp65/NF-кBp65, and catK in the femurs and tibias of obese mice.

Lycopene was shown to reduce body weight gain and improve blood glucose and lipid metabolism in obese mice, which is in line with our previous study [29]. It is known that the sustained dyslipidemia and hyperglycemia induce an increase in adipogenesis and a decrease in osteogenesis [42–44], which lead to reduced bone formation. Moreover, ROS overproduction and dyslipidemia attenuate bone material profiles and bone strength [45–47], leading to a diminished bone quality. Indeed, we found that obese mice exhibit a decreased bone biomechanical strength and a disorganized bone microstructure. Interestingly, lycopene was reported to exert bone protective effect through its antioxidant properties [24, 48]. In addition, as shown in the present study, lycopene treatment alleviates oxidative stress by increasing serum levels of T-AOC and SOD and decreasing serum levels of MDA in obese mice. The administration of lycopene for 10 weeks to obese mice results in an improvement in bone material profiles. Also, lycopene intervention increases the capacity of ultimate load in the tibias of obese mice. Together, these results suggested that lycopene has the ability of improving bone quality through the regulation of oxidative stress in obese mice.

The sustained high levels of ROS may be associated with heightened AGE expressions upon hyperglycemia stimulation [49], which further induces NF-кB activation and subsequent bone resorption [50, 51]. We demonstrated that lycopene treatment decreases the expressions of AGEs and RAGE as well as inhibits NF-кBp65 activation in the femurs and tibias of obese mice. These results were in line with the findings that lycopene is able to decrease the levels of plasma AGEs and renal RAGE [52] and suppress NF-кB activation in hyperglycemia rats and HK-2 cells [49, 52, 53]. In addition, lycopene was reported to inhibit ROS-induced NF-кB activation in pancreatic cancer cells [54]. Moreover, dietary lycopene could decrease bone resorption in postmenopausal women [26, 55]. In the present study, we demonstrated that lycopene decreases catK expression and inhibits bone resorption in obese mice. Together, these findings suggested that lycopene may improve bone quality through the regulation of the AGE/RAGE/ROS/NF-кB signaling pathway in HFD-induced obese mice.

The dyslipidemia in HFD-induced obese mice was normalized by lycopene intervention in the current study, which was associated with a reduction in AGE generation and NF-кB activation. Notably, AGE overproduction contributes to dyslipidemia through reacting with plasma lipoproteins [21]. In addition, high cholesterol may activate NF-кB signaling through promotion of ROS production [56], which accelerates osteoclastic bone resorption and consequent osteoporosis [18]. Collectively, these findings suggest that lycopene may preserve bone remodeling through the regulation of lipid metabolism and inhibition of AGE production in obese mice.

In the present study, we demonstrated that HFD-induced hyperglycemia mice exhibit an increase in the BMD of the metaphysis and a decrease in the diaphysis of the tibias. This is in line with the observations from Ho-Pham. et al. who reported that type 2 diabetes mellitus (T2DM) patients had higher trabecular BMD but lower cortical BMD [57]. It is generally accepted that patients with T2DM have normal or higher BMD in the femoral neck and spine [58, 59]. However, an elevation of BMD in T2DM is associated with increased risk of bone fragility [60]. Indeed, disorganized bone microarchitectures, such as increased cortical porosity and decreased cortical bone density, account for an increased risk of fractures [57]. In addition, the compromised bone material profiles may attenuate bone quality [61]. Here, we demonstrated that the obesity-induced hyperglycemia mice exhibit abnormal tibia remodeling characterized by deteriorated microstructure and compromised material profiles, which were prevented by lycopene treatment. However, we only evaluated the alterations of the material profiles in the whole tibia powders. In the future, we will compare the differences of bone material profiles between epiphysis, metaphysis, and diaphysis in obese mice in response to lycopene treatment. Taken together, these findings suggested that lycopene consumption may improve bone quality through the regulation of bone microstructure and material profiles.

In our study, we confirmed that the disordered glucose and lipid metabolism in obese mice were restored by metformin treatment, which is in line with the investigations from the other groups [62, 63]. In addition, the bone protective effect conferred by metformin was comparable to that of lycopene, including an improvement in the bone microstructure, biomechanical strength, and material profiles in obesity-induced hyperglycemia mice. These alterations are associated with the restorations of bone histomorphological architecture. Interestingly, Bornstein et al. reported that metformin was able to reverse the inhibition of osteoblastogenesis and to suspend bone marrow and inguinal adipose expansion in HFD-induced obese mice [64]. Notably, we demonstrated that metformin intervention inhibits an elevation of osteoclast activity and oxidative stress in obesity-induced hyperglycemia mice. In agreement with the current findings, metformin was reported to suppress osteoclast differentiation in collagen-induced arthritic mice [65] and inhibit ROS generation under osteoporotic conditions [66]. As mentioned above, an elevation of oxidative stress contributes to osteoclastic bone resorption rather than osteoblastic bone formation. Therefore, it is suggested that metformin may improve bone metabolism through the regulation of redox homeostasis. However, the underlying mechanism whereby metformin exerts antiosteoporotic effect still merits further investigation.

The current study still has limitations. We did not investigate the effect of metformin on the expression levels of the proteins involved in AGE/RAGE/NF-кB signaling pathway. However, metformin has been reported to inhibit the AGE formation [67]. In addition, a number of studies have reported that metformin could downregulate RAGE expression and reduce NF-*κ*B activation in various cells, including osteoblastic cells [68–70], MCF-7, MDA-MB-231 cells [71], and osteosarcoma stem cells [72]. Moreover, Bian et al. reported [73] that low concentration of metformin inhibited osteoclast differentiation, as well as downregulated catK expression, and its inhibitory effect was gradually enhanced with increasing concentrations of metformin. Therefore, the effects of metformin could be in dose-dependent manner and deserve to be addressed in the future study.

## 5. Conclusion

In summary, we demonstrated that lycopene improves bone quality in HFD-induced obese mice. The underlying mechanism might be associated with the regulation of the AGE/RAGE/NF-кB signaling pathway. These results suggest that dietary supplementation of lycopene may offer a new therapeutic strategy for the management of obesity and its associated-osteoporosis, which needs to be further evaluated in clinical trials.

## Figures and Tables

**Table 1:** Study design.

Group	n	Diet	Intervention
Normal control (NC)	9	Regular chow	Equal volume of sunflower oil
HFD-C	9	High-fat diet	Equal volume of sunflower oil
LYC	9	High-fat diet	Lycopene (15 mg/kg, dissolved in sunflower oil)
MET	9	High-fat diet	Metformin (500 mg/kg, dissolved in sunflower oil)

**Table 2:** The trabecular bone structural parameters analyzed by *μ*-CT.

Parameter	Full name	Meaning
BMD	Bone mineral density	Total bone mineral content divided by the total volume
BV/TV	Bone volume fraction	The ratio of the segmented bone volume to the total volume
BS/TV	Bone surface density	The bone surface area per total volume
Tb.N	Trabecular number	The number of trabecular in the region of interest
Tb.Sp	Trabecular separation	The distance between trabecular bones measured by 3D construction
Tb.Th	Trabecular thickness	The thickness of trabecular bone
Conn.D	Connectivity density	The number of connections between trabecular meshwork per cubic millimeter
SMI	Structure model index	The proportion between plate-like structure and rod-like structure in trabecular bone
Tt.Ar	Total cross-sectional area	Total area of cortical bone
Ct.Ar	Cortical bone area	Cross-sectional area of the cortical bone
Ct.Th	Cortical bone thickness	The thickness of cortical bone
Ma.Ar	Bone marrow area	Bone marrow cavity

**Table 3:** Lycopene reduces body weight gain and improves glucose and lipid metabolism in HFD-induced obese mice (*x* ± SD).

Parameters	Normal	HFD-C	HFD + MET	HFD + LYC
BW (0 week, g)	20.00 ± 2.00	20.00 ± 2.00	20.00 ± 2.00	20.00 ± 2.00
BW (12^th^ week, g)	46.37 ± 1.79	54.37 ± 7.38^#^	54.15 ± 5.94^#^	54.93 ± 4.65^#^
BW (22^nd^ week, g)	44.02 ± 2.78	66.18 ± 7.28^#^	49.29 ± 6.41^∗^	52.33 ± 7.71^∗^
TG (*μ*M)	1.02 ± 0.17	1.35 ± 0.08^#^	1.04 ± 0.08^∗^	1.13 ± 0.05^∗^
TC (*μ*M)	4.30 ± 0.36	7.6 ± 0.97^#^	6.10 ± 0.86^∗^	6.14 ± 0.84^∗^
LDL (*μ*M)	0.49 ± 0.15	2.56 ± 0.33^#^	0.90 ± 0.32^∗^	0.91 ± 0.30^∗^
HDL (*μ*M)	5.44 ± 0.97	3.83 ± 0.66^#^	5.45 ± 0.97^∗^	6.36 ± 0.90^∗^
FBG (12^th^ week, mM/L)	5.35 ± 0.98	8.13 ± 0.86	8.68 ± 2.56	8.05 ± 0.84
FBG (22^nd^ week, mM/L)	5.92 ± 0.80	9.06 ± 1.01^#^	7.23 ± 0.39^∗^	6.76 ± 0.90^∗^

Notes: BW, body weight; FBG, fasting blood glucose; TG, triglycerides; TC, total cholesterol; HDL, high-density lipoprotein; LDL, low-density lipoprotein; HFD-C, high-fat diet-fed control; MET, metformin treatment; LYC, lycopene treatment; data are presented as mean ± SD. ^#^ vs the NC group. ^∗^ vs the HFD-C group. *p* < 0.05 was considered statistical difference.

**Table 4:** The mice blood glucose levels at 20^th^ week (0, 30, 60, 90, and 120 min) (*x* ± SD).

Time (min)	Normal	HFD-C	HFD + MET	HFD + LYC
0	3.79 ± 1.49	6.28 ± 0.84^#^	4.78 ± 1.11^∗^	3.04 ± 0.30^∗^
30	10.08 ± 1.06	15.1 ± 2.99^#^	11.12 ± 4.31^∗^	11.72 ± 1.20^∗^
60	7.17 ± 1.12	11.57 ± 1.11^#^	9.26 ± 0.35^∗^	8.44 ± 0.68^∗^
90	5.77 ± 1.17	8.55 ± 0.85^#^	7.01 ± 1.87	5.38 ± 1.24^∗^
120	4.18 ± 0.87	7.66 ± 1.47^#^	6.53 ± 0.94	5.08 ± 0.94^∗^

Notes: HFD-C, high-fat diet-fed control; MET, metformin treatment; LYC, lycopene treatment; data are presented as mean ± SD. ^#^ vs the NC group. ^∗^ vs the HFD-C group. *p* < 0.05 was considered statistical difference.

**Table 5:** The correlation between serum markers and bone quality.

Parameters	TG	TC	LDL	HDL	MDA	SOD	T-AOC
BMD (*r*)	0.860	0.824	0.217	-0.344	-0.045	0.552	0.888
*p* value	0.027^#^	0.043^#^	0.680	0.505	0.932	0.256	0.018^#^
BV/TV (*r*)	-0.273	-0.146	-0.821	0.357	-0.775	0.612	0.271
*p* value	0.553	0.708	0.067	0.488	-0.041^#^	0.080	0.480
BS/TV (*r*)	-0.285	-0.105	-0.508	0.119	-0.733	0.578	0.184
*p* value	0.553	0.788	0.163	0.821	0.061	0.103	0.637
Tb.Th (*r*)	-0.027	-0.138	-0.556	0.737	-0.662	0.210	0.042
*p* value	0.953	0.723	0.120	0.095	0.095	0.587	0.587
Tb.Sp (*r*)	0.468	0.174	0.232	-0.215	0.385	-0.912	-0.215
*p* value	0.289	0.289	0.548	0.683	0.394	0.001^#^	0.579
Tb.N (*r*)	-0.155	-0.06	-0.565	0.206	-0.837	0.531	0.018
*p* value	0.738	0.872	0.113	0.113	0.019^#^	0.019^#^	0.964
Conn.D (*r*)	-0.309	-0.106	-0.206	0.369	0.354	-0.295	-0.111
*p* value	0.499	0.784	0.594	0.471	0.471	0.471	0.776
SMI (*r*)	0.527	0.398	0.096	-0.576	-0.252	0.815	0.109
*p* value	0.223	0.288	0.288	0.231	0.586	0.007^#^	0.007^#^
BMD (r)	-0.612	-0.607	-0.278	0.213	-0.804	0.679	0.560
*p* value	0.144	0.082	0.468	0.468	0.012^#^	0.012^#^	0.117
Tt.Ar (*r*)	-0.749	-0.183	-0.384	0.133	-0.498	0.027	0.216
*p* value	0.052	0.637	0.637	0.801	0.801	0.801	0.577
Ct.Ar (*r*)	0.534	0.074	0.209	-0.032	0.650	-0.013	-0.070
*p* value	0.216	0.849	0.589	0.952	0.114	0.973	0.973
Ct.Th (*r*)	-0.095	-0.352	-0.108	0.08	-0.682	0.052	0.401
*p* value	0.839	0.351	0.781	0.781	0.781	0.894	0.894
Ma.Ar (*r*)	0.847	0.372	0.452	-0.212	0.209	-0.058	-0.419
*p* value	0.016^#^	0.324	0.221	0.687	0.653	0.883	0.261
F/Ar (*r*)	-0.332	-0.251	-0.033	0.306	-0.014	0.012	0.184
*p* value	0.466	0.515	0.933	0.933	0.976	0.974	0.635

Notes: TG, triglycerides; TC, total cholesterol; HDL, high-density lipoprotein; LDL, low-density lipoprotein; HFD-C, high-fat diet-fed control; MET, metformin; LYC, lycopene; T-AOC, total antioxidant capacity; SOD, superoxide dismutase; MDA, malondialdehyde; BMD, bone mineral density; BV/TV, bone volume fraction; BS/TV, bone surface density; Tb.N, trabecular number; Tb.Sp, trabecular separation; Tb.Th, trabecular thickness; Conn.D, connectivity density; SMI, structure Model Index; Tt.Ar, total cross-sectional area; Ct.Ar, cortical area; Ct.Th, cortical thickness; Ma.Ar, marrow area. ^#^, *p* < 0.05 was considered statistical difference.

## Data Availability

The data used to support the findings of this study are available from the corresponding authors upon request.
